# Gadolinium scandium germanide, Gd_2_Sc_3_Ge_4_

**DOI:** 10.1107/S1600536809007211

**Published:** 2009-03-06

**Authors:** Sumohan Misra, Gordon J. Miller

**Affiliations:** a Department of Chemistry and Ames Laboratory, Iowa State University, Ames, IA 50011, USA

## Abstract

Gd_2_Sc_3_Ge_4_ adopts the ortho­rhom­bic Pu_5_Rh_4_-type structure. The crystal structure contains six sites in the asymmetric unit: two sites are statistically occupied by rare-earth atoms with Gd:Sc ratios of 0.967 (4):0.033 (4) and 0.031 (3):0.969 (3), one site (.*m*. symmetry) is occupied by Sc atoms, and three distinct sites (two of which with .*m*. symmetry) are occupied by Ge atoms. The rare-earth atoms form two-dimensional slabs with Ge atoms occupying the trigonal-prismatic voids.

## Related literature

The title compound adopts the Pu_5_Rh_4_-type structure (Cromer, 1977 ▶). For Ge⋯Ge distances, see: Mozharivskyj *et al.* (2003 ▶); Holtzberg *et al.* (1967 ▶); Smith *et al.* (1967 ▶). For atomic radii. see: Shannon (1976 ▶). For the Hamilton significance test, see: Hamilton (1965 ▶). For a mixed rare-earth system, see: Misra & Miller (2008 ▶).

## Experimental

### 

#### Crystal data

Gd_2_Sc_3_Ge_4_
*M* *_r_* = 739.74
Orthorhombic, 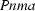

*a* = 7.2445 (13) Å
*b* = 14.101 (3) Å
*c* = 7.4930 (14) Å
*V* = 765.4 (2) Å^3^
*Z* = 4
Mo *K*α radiation
μ = 34.91 mm^−1^
*T* = 298 K
0.06 × 0.05 × 0.01 mm

#### Data collection

Bruker SMART CCD area-detector diffractometer
Absorption correction: multi-scan (*SADABS*; Bruker, 2002 ▶) *T*_min_ = 0.127, *T*_max_ = 0.705
6182 measured reflections
958 independent reflections
816 reflections with *I* > 2s(*I*)
*R*_int_ = 0.070

#### Refinement

*R*[*F* ^2^ > 2σ(*F* ^2^)] = 0.031
*wR*(*F* ^2^) = 0.066
*S* = 1.05
958 reflections
49 parameters
Δρ_max_ = 2.20 e Å^−3^
Δρ_min_ = −1.44 e Å^−3^

### 

Data collection: *SMART* (Bruker, 2002 ▶); cell refinement: *SAINT* (Bruker, 2002 ▶); data reduction: *SAINT*; program(s) used to solve structure: *SHELXS97* (Sheldrick, 2008 ▶); program(s) used to refine structure: *SHELXL97* (Sheldrick, 2008 ▶); molecular graphics: *DIAMOND* (Brandenburg, 1999 ▶); software used to prepare material for publication: *SHELXTL* (Sheldrick, 2008 ▶).

## Supplementary Material

Crystal structure: contains datablocks I, global. DOI: 10.1107/S1600536809007211/mg2062sup1.cif

Structure factors: contains datablocks I. DOI: 10.1107/S1600536809007211/mg2062Isup2.hkl

Additional supplementary materials:  crystallographic information; 3D view; checkCIF report

## Figures and Tables

**Fig. 1.**
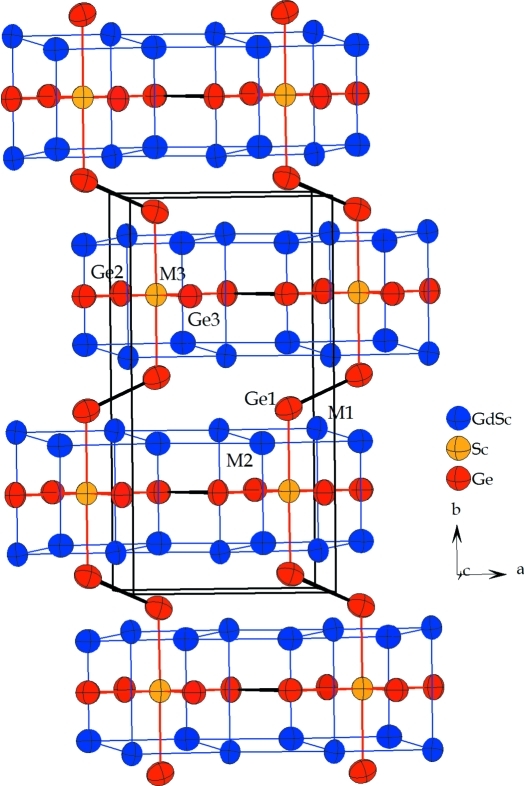
View of Gd2Sc3Ge4 along [001], with displacement ellipsoids drawn at the 99 % probability level.

## supplementary crystallographic information

### Comment

Continuing our efforts in mixed rare-earth systems bearing the formula (R_1-_*_x_*R'*_x_*)_5_T_4_, where T = Si, Ge, Ga, Sn, we have studied the effect of substitution of Gd by nonmagnetic Sc. Gd_2_Sc_3_Ge_4_ crystallizes in the orthorhombic Pu_5_Rh_4_-type structure (Cromer, 1977). Two 3^2^434 nets built up of Gd and Sc atoms are placed over one another to form two-dimensional slabs with additional Sc atoms in pseudo-cubic coordination and Ge atoms in trigonal prismatic voids (Fig. 1).

The two metal sites situated at the edges of the cube (M1 and M2) exhibit mixed site occupancies, with Gd (the larger atom; Shannon, 1976) having a preference for the M1 site and Sc (the smaller atom; Shannon, 1976) having a preference for the M2 site. The M3 site is completely occupied by Sc atom. This disordered model which introduces two additional refinement parameters yields a statistically significant improvement over various ordered models, at the 0.5% significance level according to a Hamilton's significance test on the crystallographic *R* factor (Hamilton, 1965).

The Ge1–Ge1 distances are intermediate between those in Gd_5_Ga_2_Ge_2_ [2.741 (1) Å; Gd_5_Si_4_-type (Holtzberg *et al.*, 1967)] and Gd_5_Ga_0.7_Ge_3.3_ [3.461 (5) Å; Sm_5_Ge_4_-type (Smith *et al.*, 1967)] (Mozharivskyj *et al.*, 2003).

### Experimental

Gd_2_Sc_3_Ge_4_ was prepared by arc-melting pieces of the constituent elements (Gd, 99.99 wt. %, Materials Preparation Center, Ames Laboratory; Sc, 99.99 wt. %, Materials Preparation Center, Ames Laboratory; Ge, 99.9999 wt. %, Alfa Aesar) in an argon atmosphere on a water-cooled copper hearth. The ingot had a total weight of ca. 0.8 g and was remelted six times with the button being turned over after each melting to ensure homogeneity. Weight losses during melting were less than 0.1 wt. %.

### Refinement

A disordered model works best compared to an ordered model and this has been confirmed by a Hamilton's significance test on the crystallographic *R* factor. We formulated four hypotheses to be tested: (A) M1 site is all Gd, M2 site is all Sc; (B) M1 site is all Gd, M2 site is mixed with Gd and Sc; (C) M1 site is mixed with Gd and Sc, M1 site is all Sc; and (D) both M1 and M2 sites are mixed with Gd and Sc. The number of parameters refined in the four cases were *m_A_* = 47, *m_B_* = 48, *m_C_* = 48, and *m_D_* = 49. There were 958 reflections. The *R* factors achieved were *R_A_ = *0.0333, *R_B_ = *0.0318, *R_C_ = *0.0326, and *R_D_ = *0.0313. We could reject hypotheses (A), (B), and (C) at the 0.5% level of significance corresponding to hypothesis (D).

### Crystal data

**Table:**

Gd_2_Sc_3_Ge_4_	*F*(000) = 1276
*M**_r_* = 739.74	*D*_x_ = 6.419 Mg m^−^^3^
Orthorhombic, *P**n**m**a*	Mo *K*α radiation, λ = 0.71073 Å
Hall symbol: -P 2ac 2n	Cell parameters from 6182 reflections
*a* = 7.2445 (13) Å	θ = 3.1–27.8°
*b* = 14.101 (3) Å	µ = 34.91 mm^−^^1^
*c* = 7.4930 (14) Å	*T* = 298 K
*V* = 765.4 (2) Å^3^	Plate, grey
*Z* = 4	0.06 × 0.05 × 0.01 mm

### Data collection

**Table:**

Bruker SMART CCD area-detector diffractometer	958 independent reflections
Radiation source: fine-focus sealed tube	816 reflections with *I* > 2s(*I*)
graphite	*R*_int_ = 0.070
φ and ω scans	θ_max_ = 28.2°, θ_min_ = 2.9°
Absorption correction: multi-scan (*SADABS*; Bruker, 2002)	*h* = −9→9
*T*_min_ = 0.127, *T*_max_ = 0.705	*k* = −17→18
6182 measured reflections	*l* = −9→9

### Refinement

**Table:**

Refinement on *F*^2^	Primary atom site location: structure-invariant direct methods
Least-squares matrix: full	Secondary atom site location: difference Fourier map
*R*[*F*^2^ > 2σ(*F*^2^)] = 0.031	*w* = 1/[σ^2^(*F*_o_^2^) + (0.0171*P*)^2^ + 3.8343*P*] where *P* = (*F*_o_^2^ + 2*F*_c_^2^)/3
*wR*(*F*^2^) = 0.066	(Δ/σ)_max_ = 0.001
*S* = 1.05	Δρ_max_ = 2.20 e Å^−^^3^
958 reflections	Δρ_min_ = −1.44 e Å^−^^3^
49 parameters	Extinction correction: *SHELXL97* (Bruker, 2002), Fc^*^=kFc[1+0.001xFc^2^λ^3^/sin(2θ)]^-1/4^
0 restraints	Extinction coefficient: 0.00245 (16)

### Special details

**Table:**

Geometry. All e.s.d.'s (except the e.s.d. in the dihedral angle between two l.s. planes) are estimated using the full covariance matrix. The cell e.s.d.'s are taken into account individually in the estimation of e.s.d.'s in distances, angles and torsion angles; correlations between e.s.d.'s in cell parameters are only used when they are defined by crystal symmetry. An approximate (isotropic) treatment of cell e.s.d.'s is used for estimating e.s.d.'s involving l.s. planes.
Refinement. Refinement of *F*^2^ against ALL reflections. The weighted *R*-factor *wR* and goodness of fit *S* are based on *F*^2^, conventional *R*-factors *R* are based on *F*, with *F* set to zero for negative *F*^2^. The threshold expression of *F*^2^ > σ(*F*^2^) is used only for calculating *R*-factors(gt) *etc*. and is not relevant to the choice of reflections for refinement. *R*-factors based on *F*^2^ are statistically about twice as large as those based on *F*, and *R*- factors based on ALL data will be even larger.

### Fractional atomic coordinates and isotropic or equivalent isotropic displacement parameters (Å^2^)

**Table:**

	*x*	*y*	*z*	*U*_iso_*/*U*_eq_	Occ. (<1)
Gd1	0.99788 (5)	0.40395 (3)	0.17641 (5)	0.00746 (16)	0.967 (4)
Sc1	0.99788 (5)	0.40395 (3)	0.17641 (5)	0.00746 (16)	0.033 (4)
Gd2	0.6602 (2)	0.37594 (9)	0.83229 (17)	0.0081 (5)	0.031 (3)
Sc2	0.6602 (2)	0.37594 (9)	0.83229 (17)	0.0081 (5)	0.969 (3)
Sc3	0.1761 (3)	0.7500	0.5005 (3)	0.0072 (4)	
Ge1	0.82216 (12)	0.45888 (6)	0.54068 (11)	0.0091 (2)	
Ge2	0.03997 (16)	0.7500	0.12561 (16)	0.0081 (3)	
Ge3	0.30680 (16)	0.7500	0.86322 (15)	0.0081 (3)	

### Atomic displacement parameters (Å^2^)

**Table:**

	*U*^11^	*U*^22^	*U*^33^	*U*^12^	*U*^13^	*U*^23^
Gd1	0.0064 (2)	0.0085 (2)	0.0074 (2)	0.00025 (14)	0.00024 (15)	0.00013 (15)
Sc1	0.0064 (2)	0.0085 (2)	0.0074 (2)	0.00025 (14)	0.00024 (15)	0.00013 (15)
Gd2	0.0095 (8)	0.0083 (7)	0.0065 (8)	0.0000 (5)	0.0006 (5)	0.0001 (5)
Sc2	0.0095 (8)	0.0083 (7)	0.0065 (8)	0.0000 (5)	0.0006 (5)	0.0001 (5)
Sc3	0.0067 (10)	0.0079 (10)	0.0072 (10)	0.000	0.0000 (8)	0.000
Ge1	0.0113 (5)	0.0086 (4)	0.0073 (4)	0.0011 (3)	−0.0009 (3)	0.0008 (3)
Ge2	0.0068 (6)	0.0098 (6)	0.0077 (6)	0.000	0.0012 (5)	0.000
Ge3	0.0085 (6)	0.0082 (6)	0.0077 (6)	0.000	−0.0008 (5)	0.000

### Geometric parameters (Å, °)

**Table:**

Gd1—Ge3^i^	2.9452 (9)	Sc3—Gd2^xiv^	3.268 (2)
Gd1—Ge1^ii^	2.9605 (10)	Sc3—Sc2^xii^	3.283 (2)
Gd1—Ge1	3.1097 (10)	Sc3—Gd2^iii^	3.283 (2)
Gd1—Ge3^iii^	3.1101 (10)	Ge1—Sc2^i^	2.8070 (16)
Gd1—Ge2^iv^	3.1478 (10)	Ge1—Gd2^i^	2.8070 (16)
Gd1—Ge1^v^	3.1519 (10)	Ge1—Sc2^xv^	2.8759 (17)
Gd1—Ge1^i^	3.1861 (10)	Ge1—Gd2^xv^	2.8759 (17)
Gd1—Sc3^i^	3.4681 (17)	Ge1—Ge1^v^	2.8907 (17)
Gd1—Sc3^iii^	3.4877 (17)	Ge1—Gd1^xvi^	2.9605 (10)
Gd1—Sc2^i^	3.5083 (15)	Ge1—Sc1^xvi^	2.9605 (10)
Gd1—Gd2^i^	3.5083 (15)	Ge1—Sc3^iii^	2.9614 (10)
Gd1—Gd2^vi^	3.5762 (15)	Ge2—Ge3^vi^	2.7572 (17)
Gd2—Ge1	2.7421 (15)	Ge2—Sc2^xiv^	2.7668 (16)
Gd2—Ge2^vii^	2.7668 (16)	Ge2—Gd2^xiv^	2.7668 (16)
Gd2—Ge1^viii^	2.8070 (16)	Ge2—Sc2^xiii^	2.7668 (16)
Gd2—Ge2^iii^	2.8234 (17)	Ge2—Gd2^xiii^	2.7668 (16)
Gd2—Ge1^ix^	2.8759 (17)	Ge2—Sc3^xvi^	2.800 (2)
Gd2—Ge3^x^	2.9010 (16)	Ge2—Sc2^xii^	2.8234 (17)
Gd2—Sc3^vii^	3.268 (2)	Ge2—Gd2^xii^	2.8234 (17)
Gd2—Sc3^iii^	3.283 (2)	Ge2—Gd2^iii^	2.8234 (17)
Gd2—Gd1^viii^	3.5083 (15)	Ge2—Sc2^iii^	2.8234 (17)
Gd2—Sc1^viii^	3.5083 (15)	Ge2—Sc1^iv^	3.1478 (10)
Gd2—Sc2^xi^	3.552 (3)	Ge3—Ge2^xvii^	2.7572 (17)
Gd2—Gd2^xi^	3.552 (3)	Ge3—Sc3^xv^	2.864 (2)
Sc3—Ge2^ii^	2.800 (2)	Ge3—Sc2^xviii^	2.9010 (16)
Sc3—Ge3^ix^	2.864 (2)	Ge3—Gd2^xviii^	2.9010 (16)
Sc3—Ge3	2.878 (2)	Ge3—Sc2^x^	2.9010 (16)
Sc3—Ge1^xii^	2.9614 (10)	Ge3—Gd2^x^	2.9010 (16)
Sc3—Ge1^iii^	2.9614 (10)	Ge3—Gd1^xix^	2.9452 (9)
Sc3—Ge2	2.977 (2)	Ge3—Sc1^xix^	2.9452 (9)
Sc3—Sc2^xiii^	3.268 (2)	Ge3—Gd1^viii^	2.9452 (9)
Sc3—Gd2^xiii^	3.268 (2)	Ge3—Sc1^viii^	2.9452 (9)
Sc3—Sc2^xiv^	3.268 (2)	Ge3—Gd1^iii^	3.1101 (10)
			
Ge3^i^—Gd1—Ge1^ii^	94.17 (3)	Ge3^ix^—Sc3—Gd2^iii^	127.47 (6)
Ge3^i^—Gd1—Ge1	87.88 (3)	Ge3—Sc3—Gd2^iii^	126.76 (6)
Ge1^ii^—Gd1—Ge1	137.90 (3)	Ge1^xii^—Sc3—Gd2^iii^	117.23 (7)
Ge3^i^—Gd1—Ge3^iii^	82.64 (2)	Ge1^iii^—Sc3—Gd2^iii^	51.79 (3)
Ge1^ii^—Gd1—Ge3^iii^	133.89 (3)	Ge2—Sc3—Gd2^iii^	53.35 (4)
Ge1—Gd1—Ge3^iii^	88.12 (3)	Sc2^xiii^—Sc3—Gd2^iii^	71.49 (4)
Ge3^i^—Gd1—Ge2^iv^	82.83 (3)	Gd2^xiii^—Sc3—Gd2^iii^	71.49 (4)
Ge1^ii^—Gd1—Ge2^iv^	81.64 (3)	Sc2^xiv^—Sc3—Gd2^iii^	105.69 (6)
Ge1—Gd1—Ge2^iv^	140.09 (3)	Gd2^xiv^—Sc3—Gd2^iii^	105.69 (6)
Ge3^iii^—Gd1—Ge2^iv^	52.28 (3)	Sc2^xii^—Sc3—Gd2^iii^	65.50 (6)
Ge3^i^—Gd1—Ge1^v^	86.21 (3)	Gd2—Ge1—Sc2^i^	144.75 (4)
Ge1^ii^—Gd1—Ge1^v^	83.153 (17)	Gd2—Ge1—Gd2^i^	144.75 (4)
Ge1—Gd1—Ge1^v^	54.98 (3)	Sc2^i^—Ge1—Gd2^i^	0.00 (5)
Ge3^iii^—Gd1—Ge1^v^	141.82 (3)	Gd2—Ge1—Sc2^xv^	85.83 (4)
Ge2^iv^—Gd1—Ge1^v^	160.52 (3)	Sc2^i^—Ge1—Sc2^xv^	118.86 (4)
Ge3^i^—Gd1—Ge1^i^	161.59 (3)	Gd2^i^—Ge1—Sc2^xv^	118.86 (4)
Ge1^ii^—Gd1—Ge1^i^	104.08 (3)	Gd2—Ge1—Gd2^xv^	85.83 (4)
Ge1—Gd1—Ge1^i^	80.275 (17)	Sc2^i^—Ge1—Gd2^xv^	118.86 (4)
Ge3^iii^—Gd1—Ge1^i^	82.92 (3)	Gd2^i^—Ge1—Gd2^xv^	118.86 (4)
Ge2^iv^—Gd1—Ge1^i^	97.23 (3)	Sc2^xv^—Ge1—Gd2^xv^	0.00 (7)
Ge1^v^—Gd1—Ge1^i^	98.23 (3)	Gd2—Ge1—Ge1^v^	136.10 (5)
Ge3^i^—Gd1—Sc3^i^	52.57 (4)	Sc2^i^—Ge1—Ge1^v^	60.61 (4)
Ge1^ii^—Gd1—Sc3^i^	54.16 (3)	Gd2^i^—Ge1—Ge1^v^	60.61 (4)
Ge1—Gd1—Sc3^i^	140.20 (4)	Sc2^xv^—Ge1—Ge1^v^	58.26 (4)
Ge3^iii^—Gd1—Sc3^i^	90.58 (3)	Gd2^xv^—Ge1—Ge1^v^	58.26 (4)
Ge2^iv^—Gd1—Sc3^i^	49.79 (4)	Gd2—Ge1—Gd1^xvi^	89.24 (4)
Ge1^v^—Gd1—Sc3^i^	110.99 (4)	Sc2^i^—Ge1—Gd1^xvi^	86.99 (4)
Ge1^i^—Gd1—Sc3^i^	138.95 (4)	Gd2^i^—Ge1—Gd1^xvi^	86.99 (4)
Ge3^i^—Gd1—Sc3^iii^	52.04 (4)	Sc2^xv^—Ge1—Gd1^xvi^	138.69 (4)
Ge1^ii^—Gd1—Sc3^iii^	146.21 (4)	Gd2^xv^—Ge1—Gd1^xvi^	138.69 (4)
Ge1—Gd1—Sc3^iii^	52.97 (3)	Ge1^v^—Ge1—Gd1^xvi^	134.14 (5)
Ge3^iii^—Gd1—Sc3^iii^	51.34 (4)	Gd2—Ge1—Sc1^xvi^	89.24 (4)
Ge2^iv^—Gd1—Sc3^iii^	92.20 (3)	Sc2^i^—Ge1—Sc1^xvi^	86.99 (4)
Ge1^v^—Gd1—Sc3^iii^	93.70 (4)	Gd2^i^—Ge1—Sc1^xvi^	86.99 (4)
Ge1^i^—Gd1—Sc3^iii^	109.66 (4)	Sc2^xv^—Ge1—Sc1^xvi^	138.69 (4)
Sc3^i^—Gd1—Sc3^iii^	96.92 (4)	Gd2^xv^—Ge1—Sc1^xvi^	138.69 (4)
Ge3^i^—Gd1—Sc2^i^	130.59 (3)	Ge1^v^—Ge1—Sc1^xvi^	134.14 (5)
Ge1^ii^—Gd1—Sc2^i^	102.15 (3)	Gd1^xvi^—Ge1—Sc1^xvi^	0.000 (17)
Ge1—Gd1—Sc2^i^	49.75 (3)	Gd2—Ge1—Sc3^iii^	70.16 (5)
Ge3^iii^—Gd1—Sc2^i^	114.68 (3)	Sc2^i^—Ge1—Sc3^iii^	140.10 (6)
Ge2^iv^—Gd1—Sc2^i^	145.18 (3)	Gd2^i^—Ge1—Sc3^iii^	140.10 (6)
Ge1^v^—Gd1—Sc2^i^	50.82 (3)	Sc2^xv^—Ge1—Sc3^iii^	68.07 (5)
Ge1^i^—Gd1—Sc2^i^	48.06 (3)	Gd2^xv^—Ge1—Sc3^iii^	68.07 (5)
Sc3^i^—Gd1—Sc2^i^	154.50 (3)	Ge1^v^—Ge1—Sc3^iii^	111.92 (6)
Sc3^iii^—Gd1—Sc2^i^	101.62 (3)	Gd1^xvi^—Ge1—Sc3^iii^	71.70 (5)
Ge3^i^—Gd1—Gd2^i^	130.59 (3)	Sc1^xvi^—Ge1—Sc3^iii^	71.70 (5)
Ge1^ii^—Gd1—Gd2^i^	102.15 (3)	Gd2—Ge1—Gd1	140.21 (4)
Ge1—Gd1—Gd2^i^	49.75 (3)	Sc2^i^—Ge1—Gd1	72.53 (3)
Ge3^iii^—Gd1—Gd2^i^	114.68 (3)	Gd2^i^—Ge1—Gd1	72.53 (3)
Ge2^iv^—Gd1—Gd2^i^	145.18 (3)	Sc2^xv^—Ge1—Gd1	80.83 (4)
Ge1^v^—Gd1—Gd2^i^	50.82 (3)	Gd2^xv^—Ge1—Gd1	80.83 (4)
Ge1^i^—Gd1—Gd2^i^	48.06 (3)	Ge1^v^—Ge1—Gd1	63.25 (3)
Sc3^i^—Gd1—Gd2^i^	154.50 (3)	Gd1^xvi^—Ge1—Gd1	77.14 (2)
Sc3^iii^—Gd1—Gd2^i^	101.62 (3)	Sc1^xvi^—Ge1—Gd1	77.14 (2)
Sc2^i^—Gd1—Gd2^i^	0.00 (4)	Sc3^iii^—Ge1—Gd1	70.08 (4)
Ge3^i^—Gd1—Gd2^vi^	126.15 (3)	Ge3^vi^—Ge2—Sc2^xiv^	140.05 (3)
Ge1^ii^—Gd1—Gd2^vi^	100.11 (3)	Ge3^vi^—Ge2—Gd2^xiv^	140.05 (3)
Ge1—Gd1—Gd2^vi^	112.35 (3)	Sc2^xiv^—Ge2—Gd2^xiv^	0.00 (5)
Ge3^iii^—Gd1—Gd2^vi^	50.84 (3)	Ge3^vi^—Ge2—Sc2^xiii^	140.05 (3)
Ge2^iv^—Gd1—Gd2^vi^	49.15 (3)	Sc2^xiv^—Ge2—Sc2^xiii^	79.86 (7)
Ge1^v^—Gd1—Gd2^vi^	146.68 (3)	Gd2^xiv^—Ge2—Sc2^xiii^	79.86 (7)
Ge1^i^—Gd1—Gd2^vi^	48.63 (3)	Ge3^vi^—Ge2—Gd2^xiii^	140.05 (3)
Sc3^i^—Gd1—Gd2^vi^	97.04 (4)	Sc2^xiv^—Ge2—Gd2^xiii^	79.86 (7)
Sc3^iii^—Gd1—Gd2^vi^	100.63 (4)	Gd2^xiv^—Ge2—Gd2^xiii^	79.86 (7)
Sc2^i^—Gd1—Gd2^vi^	96.57 (3)	Sc2^xiii^—Ge2—Gd2^xiii^	0.00 (5)
Gd2^i^—Gd1—Gd2^vi^	96.57 (3)	Ge3^vi^—Ge2—Sc3^xvi^	114.80 (6)
Ge1—Gd2—Ge2^vii^	92.98 (5)	Sc2^xiv^—Ge2—Sc3^xvi^	72.27 (5)
Ge1—Gd2—Ge1^viii^	94.01 (4)	Gd2^xiv^—Ge2—Sc3^xvi^	72.27 (5)
Ge2^vii^—Gd2—Ge1^viii^	150.21 (6)	Sc2^xiii^—Ge2—Sc3^xvi^	72.27 (5)
Ge1—Gd2—Ge2^iii^	91.61 (5)	Gd2^xiii^—Ge2—Sc3^xvi^	72.27 (5)
Ge2^vii^—Gd2—Ge2^iii^	93.56 (4)	Ge3^vi^—Ge2—Sc2^xii^	62.63 (4)
Ge1^viii^—Gd2—Ge2^iii^	115.13 (5)	Sc2^xiv^—Ge2—Sc2^xii^	86.39 (4)
Ge1—Gd2—Ge1^ix^	117.05 (5)	Gd2^xiv^—Ge2—Sc2^xii^	86.39 (4)
Ge2^vii^—Gd2—Ge1^ix^	90.00 (5)	Sc2^xiii^—Ge2—Sc2^xii^	138.11 (5)
Ge1^viii^—Gd2—Ge1^ix^	61.14 (4)	Gd2^xiii^—Ge2—Sc2^xii^	138.11 (5)
Ge2^iii^—Gd2—Ge1^ix^	150.92 (6)	Sc3^xvi^—Ge2—Sc2^xii^	139.64 (4)
Ge1—Gd2—Ge3^x^	148.48 (6)	Ge3^vi^—Ge2—Gd2^xii^	62.63 (4)
Ge2^vii^—Gd2—Ge3^x^	95.19 (5)	Sc2^xiv^—Ge2—Gd2^xii^	86.39 (4)
Ge1^viii^—Gd2—Ge3^x^	93.83 (5)	Gd2^xiv^—Ge2—Gd2^xii^	86.39 (4)
Ge2^iii^—Gd2—Ge3^x^	57.57 (4)	Sc2^xiii^—Ge2—Gd2^xii^	138.11 (5)
Ge1^ix^—Gd2—Ge3^x^	93.37 (5)	Gd2^xiii^—Ge2—Gd2^xii^	138.11 (5)
Ge1—Gd2—Sc3^vii^	149.10 (6)	Sc3^xvi^—Ge2—Gd2^xii^	139.64 (4)
Ge2^vii^—Gd2—Sc3^vii^	58.41 (5)	Sc2^xii^—Ge2—Gd2^xii^	0.00 (7)
Ge1^viii^—Gd2—Sc3^vii^	105.69 (5)	Ge3^vi^—Ge2—Gd2^iii^	62.63 (4)
Ge2^iii^—Gd2—Sc3^vii^	100.88 (5)	Sc2^xiv^—Ge2—Gd2^iii^	138.11 (5)
Ge1^ix^—Gd2—Sc3^vii^	57.21 (4)	Gd2^xiv^—Ge2—Gd2^iii^	138.11 (5)
Ge3^x^—Gd2—Sc3^vii^	54.94 (5)	Sc2^xiii^—Ge2—Gd2^iii^	86.39 (4)
Ge1—Gd2—Sc3^iii^	58.06 (4)	Gd2^xiii^—Ge2—Gd2^iii^	86.39 (4)
Ge2^vii^—Gd2—Sc3^iii^	54.33 (5)	Sc3^xvi^—Ge2—Gd2^iii^	139.64 (4)
Ge1^viii^—Gd2—Sc3^iii^	148.75 (6)	Sc2^xii^—Ge2—Gd2^iii^	77.95 (6)
Ge2^iii^—Gd2—Sc3^iii^	57.77 (5)	Gd2^xii^—Ge2—Gd2^iii^	77.95 (6)
Ge1^ix^—Gd2—Sc3^iii^	141.16 (6)	Ge3^vi^—Ge2—Sc2^iii^	62.63 (4)
Ge3^x^—Gd2—Sc3^iii^	103.67 (5)	Sc2^xiv^—Ge2—Sc2^iii^	138.11 (5)
Sc3^vii^—Gd2—Sc3^iii^	105.56 (5)	Gd2^xiv^—Ge2—Sc2^iii^	138.11 (5)
Ge1—Gd2—Gd1^viii^	59.81 (3)	Sc2^xiii^—Ge2—Sc2^iii^	86.39 (4)
Ge2^vii^—Gd2—Gd1^viii^	102.14 (4)	Gd2^xiii^—Ge2—Sc2^iii^	86.39 (4)
Ge1^viii^—Gd2—Gd1^viii^	57.73 (3)	Sc3^xvi^—Ge2—Sc2^iii^	139.64 (4)
Ge2^iii^—Gd2—Gd1^viii^	147.65 (5)	Sc2^xii^—Ge2—Sc2^iii^	77.95 (6)
Ge1^ix^—Gd2—Gd1^viii^	58.16 (3)	Gd2^xii^—Ge2—Sc2^iii^	77.95 (6)
Ge3^x^—Gd2—Gd1^viii^	146.13 (5)	Gd2^iii^—Ge2—Sc2^iii^	0.00 (7)
Sc3^vii^—Gd2—Gd1^viii^	111.45 (5)	Ge3^vi^—Ge2—Sc3	116.13 (6)
Sc3^iii^—Gd2—Gd1^viii^	110.10 (4)	Sc2^xiv^—Ge2—Sc3	69.24 (5)
Ge1—Gd2—Sc1^viii^	59.81 (3)	Gd2^xiv^—Ge2—Sc3	69.24 (5)
Ge2^vii^—Gd2—Sc1^viii^	102.14 (4)	Sc2^xiii^—Ge2—Sc3	69.24 (5)
Ge1^viii^—Gd2—Sc1^viii^	57.73 (3)	Gd2^xiii^—Ge2—Sc3	69.24 (5)
Ge2^iii^—Gd2—Sc1^viii^	147.65 (5)	Sc3^xvi^—Ge2—Sc3	129.07 (7)
Ge1^ix^—Gd2—Sc1^viii^	58.16 (3)	Sc2^xii^—Ge2—Sc3	68.88 (5)
Ge3^x^—Gd2—Sc1^viii^	146.13 (5)	Gd2^xii^—Ge2—Sc3	68.88 (5)
Sc3^vii^—Gd2—Sc1^viii^	111.45 (5)	Gd2^iii^—Ge2—Sc3	68.88 (5)
Sc3^iii^—Gd2—Sc1^viii^	110.10 (4)	Sc2^iii^—Ge2—Sc3	68.88 (5)
Gd1^viii^—Gd2—Sc1^viii^	0.000 (12)	Ge3^vi^—Ge2—Sc1^iv^	63.16 (3)
Ge1—Gd2—Sc2^xi^	115.24 (3)	Sc2^xiv^—Ge2—Sc1^iv^	143.07 (5)
Ge2^vii^—Gd2—Sc2^xi^	50.07 (3)	Gd2^xiv^—Ge2—Sc1^iv^	143.07 (5)
Ge1^viii^—Gd2—Sc2^xi^	146.07 (3)	Sc2^xiii^—Ge2—Sc1^iv^	85.07 (3)
Ge2^iii^—Gd2—Sc2^xi^	51.02 (3)	Gd2^xiii^—Ge2—Sc1^iv^	85.07 (3)
Ge1^ix^—Gd2—Sc2^xi^	113.99 (3)	Sc3^xvi^—Ge2—Sc1^iv^	71.06 (4)
Ge3^x^—Gd2—Sc2^xi^	52.25 (3)	Sc2^xii^—Ge2—Sc1^iv^	125.53 (5)
Sc3^vii^—Gd2—Sc2^xi^	57.08 (3)	Gd2^xii^—Ge2—Sc1^iv^	125.53 (5)
Sc3^iii^—Gd2—Sc2^xi^	57.25 (3)	Gd2^iii^—Ge2—Sc1^iv^	73.36 (3)
Gd1^viii^—Gd2—Sc2^xi^	152.21 (2)	Sc2^iii^—Ge2—Sc1^iv^	73.36 (3)
Sc1^viii^—Gd2—Sc2^xi^	152.21 (2)	Sc3—Ge2—Sc1^iv^	135.00 (2)
Ge1—Gd2—Gd2^xi^	115.24 (3)	Ge2^xvii^—Ge3—Sc3^xv^	113.62 (6)
Ge2^vii^—Gd2—Gd2^xi^	50.07 (3)	Ge2^xvii^—Ge3—Sc3	116.29 (7)
Ge1^viii^—Gd2—Gd2^xi^	146.07 (3)	Sc3^xv^—Ge3—Sc3	130.10 (7)
Ge2^iii^—Gd2—Gd2^xi^	51.02 (3)	Ge2^xvii^—Ge3—Sc2^xviii^	59.80 (4)
Ge1^ix^—Gd2—Gd2^xi^	113.99 (3)	Sc3^xv^—Ge3—Sc2^xviii^	69.05 (5)
Ge3^x^—Gd2—Gd2^xi^	52.25 (3)	Sc3—Ge3—Sc2^xviii^	140.34 (4)
Sc3^vii^—Gd2—Gd2^xi^	57.08 (3)	Ge2^xvii^—Ge3—Gd2^xviii^	59.80 (4)
Sc3^iii^—Gd2—Gd2^xi^	57.25 (3)	Sc3^xv^—Ge3—Gd2^xviii^	69.05 (5)
Gd1^viii^—Gd2—Gd2^xi^	152.21 (2)	Sc3—Ge3—Gd2^xviii^	140.34 (4)
Sc1^viii^—Gd2—Gd2^xi^	152.21 (2)	Sc2^xviii^—Ge3—Gd2^xviii^	0.00 (3)
Sc2^xi^—Gd2—Gd2^xi^	0.00 (6)	Ge2^xvii^—Ge3—Sc2^x^	59.80 (4)
Ge2^ii^—Sc3—Ge3^ix^	178.82 (9)	Sc3^xv^—Ge3—Sc2^x^	69.05 (5)
Ge2^ii^—Sc3—Ge3	90.52 (7)	Sc3—Ge3—Sc2^x^	140.34 (4)
Ge3^ix^—Sc3—Ge3	88.30 (6)	Sc2^xviii^—Ge3—Sc2^x^	75.49 (6)
Ge2^ii^—Sc3—Ge1^xii^	87.76 (4)	Gd2^xviii^—Ge3—Sc2^x^	75.49 (6)
Ge3^ix^—Sc3—Ge1^xii^	92.35 (4)	Ge2^xvii^—Ge3—Gd2^x^	59.80 (4)
Ge3—Sc3—Ge1^xii^	95.56 (4)	Sc3^xv^—Ge3—Gd2^x^	69.05 (5)
Ge2^ii^—Sc3—Ge1^iii^	87.76 (4)	Sc3—Ge3—Gd2^x^	140.34 (4)
Ge3^ix^—Sc3—Ge1^iii^	92.35 (4)	Sc2^xviii^—Ge3—Gd2^x^	75.49 (6)
Ge3—Sc3—Ge1^iii^	95.56 (4)	Gd2^xviii^—Ge3—Gd2^x^	75.49 (6)
Ge1^xii^—Sc3—Ge1^iii^	168.04 (8)	Sc2^x^—Ge3—Gd2^x^	0.00 (4)
Ge2^ii^—Sc3—Ge2	89.64 (6)	Ge2^xvii^—Ge3—Gd1^xix^	132.513 (19)
Ge3^ix^—Sc3—Ge2	91.54 (7)	Sc3^xv^—Ge3—Gd1^xix^	73.78 (4)
Ge3—Sc3—Ge2	179.85 (9)	Sc3—Ge3—Gd1^xix^	73.09 (4)
Ge1^xii^—Sc3—Ge2	84.44 (4)	Sc2^xviii^—Ge3—Gd1^xix^	83.28 (3)
Ge1^iii^—Sc3—Ge2	84.44 (4)	Gd2^xviii^—Ge3—Gd1^xix^	83.28 (3)
Ge2^ii^—Sc3—Sc2^xiii^	124.88 (6)	Sc2^x^—Ge3—Gd1^xix^	141.76 (5)
Ge3^ix^—Sc3—Sc2^xiii^	56.01 (5)	Gd2^x^—Ge3—Gd1^xix^	141.76 (5)
Ge3—Sc3—Sc2^xiii^	127.54 (6)	Ge2^xvii^—Ge3—Sc1^xix^	132.513 (19)
Ge1^xii^—Sc3—Sc2^xiii^	120.23 (7)	Sc3^xv^—Ge3—Sc1^xix^	73.78 (4)
Ge1^iii^—Sc3—Sc2^xiii^	54.72 (4)	Sc3—Ge3—Sc1^xix^	73.09 (4)
Ge2—Sc3—Sc2^xiii^	52.35 (4)	Sc2^xviii^—Ge3—Sc1^xix^	83.28 (3)
Ge2^ii^—Sc3—Gd2^xiii^	124.88 (6)	Gd2^xviii^—Ge3—Sc1^xix^	83.28 (3)
Ge3^ix^—Sc3—Gd2^xiii^	56.01 (5)	Sc2^x^—Ge3—Sc1^xix^	141.76 (5)
Ge3—Sc3—Gd2^xiii^	127.54 (6)	Gd2^x^—Ge3—Sc1^xix^	141.76 (5)
Ge1^xii^—Sc3—Gd2^xiii^	120.23 (7)	Gd1^xix^—Ge3—Sc1^xix^	0.000 (17)
Ge1^iii^—Sc3—Gd2^xiii^	54.72 (4)	Ge2^xvii^—Ge3—Gd1^viii^	132.513 (19)
Ge2—Sc3—Gd2^xiii^	52.35 (4)	Sc3^xv^—Ge3—Gd1^viii^	73.78 (4)
Sc2^xiii^—Sc3—Gd2^xiii^	0.00 (3)	Sc3—Ge3—Gd1^viii^	73.09 (4)
Ge2^ii^—Sc3—Sc2^xiv^	124.88 (6)	Sc2^xviii^—Ge3—Gd1^viii^	141.76 (5)
Ge3^ix^—Sc3—Sc2^xiv^	56.01 (5)	Gd2^xviii^—Ge3—Gd1^viii^	141.76 (5)
Ge3—Sc3—Sc2^xiv^	127.54 (6)	Sc2^x^—Ge3—Gd1^viii^	83.28 (3)
Ge1^xii^—Sc3—Sc2^xiv^	54.72 (4)	Gd2^x^—Ge3—Gd1^viii^	83.28 (3)
Ge1^iii^—Sc3—Sc2^xiv^	120.23 (7)	Gd1^xix^—Ge3—Gd1^viii^	94.96 (4)
Ge2—Sc3—Sc2^xiv^	52.35 (4)	Sc1^xix^—Ge3—Gd1^viii^	94.96 (4)
Sc2^xiii^—Sc3—Sc2^xiv^	65.84 (6)	Ge2^xvii^—Ge3—Sc1^viii^	132.513 (19)
Gd2^xiii^—Sc3—Sc2^xiv^	65.84 (6)	Sc3^xv^—Ge3—Sc1^viii^	73.78 (4)
Ge2^ii^—Sc3—Gd2^xiv^	124.88 (6)	Sc3—Ge3—Sc1^viii^	73.09 (4)
Ge3^ix^—Sc3—Gd2^xiv^	56.01 (5)	Sc2^xviii^—Ge3—Sc1^viii^	141.76 (5)
Ge3—Sc3—Gd2^xiv^	127.54 (6)	Gd2^xviii^—Ge3—Sc1^viii^	141.76 (5)
Ge1^xii^—Sc3—Gd2^xiv^	54.72 (4)	Sc2^x^—Ge3—Sc1^viii^	83.28 (3)
Ge1^iii^—Sc3—Gd2^xiv^	120.23 (7)	Gd2^x^—Ge3—Sc1^viii^	83.28 (3)
Ge2—Sc3—Gd2^xiv^	52.35 (4)	Gd1^xix^—Ge3—Sc1^viii^	94.96 (4)
Sc2^xiii^—Sc3—Gd2^xiv^	65.84 (6)	Sc1^xix^—Ge3—Sc1^viii^	94.96 (4)
Gd2^xiii^—Sc3—Gd2^xiv^	65.84 (6)	Gd1^viii^—Ge3—Sc1^viii^	0.000 (17)
Sc2^xiv^—Sc3—Gd2^xiv^	0.00 (3)	Ge2^xvii^—Ge3—Gd1^iii^	64.56 (3)
Ge2^ii^—Sc3—Sc2^xii^	53.40 (4)	Sc3^xv^—Ge3—Gd1^iii^	134.19 (2)
Ge3^ix^—Sc3—Sc2^xii^	127.47 (6)	Sc3—Ge3—Gd1^iii^	71.12 (4)
Ge3—Sc3—Sc2^xii^	126.76 (6)	Sc2^xviii^—Ge3—Gd1^iii^	124.12 (5)
Ge1^xii^—Sc3—Sc2^xii^	51.79 (3)	Gd2^xviii^—Ge3—Gd1^iii^	124.12 (5)
Ge1^iii^—Sc3—Sc2^xii^	117.23 (7)	Sc2^x^—Ge3—Gd1^iii^	72.92 (3)
Ge2—Sc3—Sc2^xii^	53.35 (4)	Gd2^x^—Ge3—Gd1^iii^	72.92 (3)
Sc2^xiii^—Sc3—Sc2^xii^	105.69 (6)	Gd1^xix^—Ge3—Gd1^iii^	144.10 (4)
Gd2^xiii^—Sc3—Sc2^xii^	105.69 (6)	Sc1^xix^—Ge3—Gd1^iii^	144.10 (4)
Sc2^xiv^—Sc3—Sc2^xii^	71.49 (4)	Gd1^viii^—Ge3—Gd1^iii^	77.357 (18)
Gd2^xiv^—Sc3—Sc2^xii^	71.49 (4)	Sc1^viii^—Ge3—Gd1^iii^	77.357 (18)
Ge2^ii^—Sc3—Gd2^iii^	53.40 (4)		

Symmetry codes: (i) −*x*+3/2, −*y*+1, *z*−1/2; (ii) *x*+1/2, *y*, −*z*+1/2; (iii) −*x*+1, −*y*+1, −*z*+1; (iv) −*x*+1, −*y*+1, −*z*; (v) −*x*+2, −*y*+1, −*z*+1; (vi) *x*, *y*, *z*−1; (vii) −*x*+1/2, −*y*+1, *z*+1/2; (viii) −*x*+3/2, −*y*+1, *z*+1/2; (ix) *x*−1/2, *y*, −*z*+3/2; (x) −*x*+1, −*y*+1, −*z*+2; (xi) *x*, −*y*+1/2, *z*; (xii) −*x*+1, *y*+1/2, −*z*+1; (xiii) −*x*+1/2, −*y*+1, *z*−1/2; (xiv) −*x*+1/2, *y*+1/2, *z*−1/2; (xv) *x*+1/2, *y*, −*z*+3/2; (xvi) *x*−1/2, *y*, −*z*+1/2; (xvii) *x*, *y*, *z*+1; (xviii) −*x*+1, *y*+1/2, −*z*+2; (xix) −*x*+3/2, *y*+1/2, *z*+1/2.
